# Supplementary material for: Neuroprotective and Anti-Amyloid β Effect and Main Chemical Profiles of White Tea: Comparison Against Green, Oolong and Black Tea
Source: Molecules. 2019 May 19;24(10):1926. doi: 10.3390/molecules24101926 (PMC6571989; doi:10.3390/molecules24101926)
Supplement: Supplementary file 1 [file molecules-24-01926-s001.pdf]

molecules

Supplemental Materials

Neuroprotective and Anti-Amyloid β Effect and Main Chemical Profiles of White Tea: Comparison Against Green, Oolong and Black Tea

Xinlei Li[1, 2, 3], Scott D. Smid[3], Jun Lin[2,4], Zhihong Gong[2], Chen Si[1, 2], Fangning You[1], Yan Zhang[1], Zhilong Hao[1, 2], Hongzheng Lin[1], Xiaomin Yu[2*], and Xinyi Jin[1*]

1 College of Horticulture, Fujian Agriculture and Forestry University, Fuzhou 350002, China

2 FAFU-UCR Joint Center for Horticultural Biology and Metabolomics, Fujian Provincial Key Laboratory of Haixia Applied Plant Systems Biology, Fujian Agriculture and Forestry University, Fuzhou 350002, China

3 Discipline of Pharmacology, School of Medical Sciences, Faculty of Health Sciences, The University of Adelaide, South Australia 5000, Australia

4 Fujian Institute of Microbiology, Fuzhou 350007, China

* Correspondence: (X.Y.): xmyu0616@fafu.edu.cn ; Tel.: +86-591-8639-1591;
(X.J.): jxy427@fafu.edu.cn ; Tel.: +86-136-7501-4927

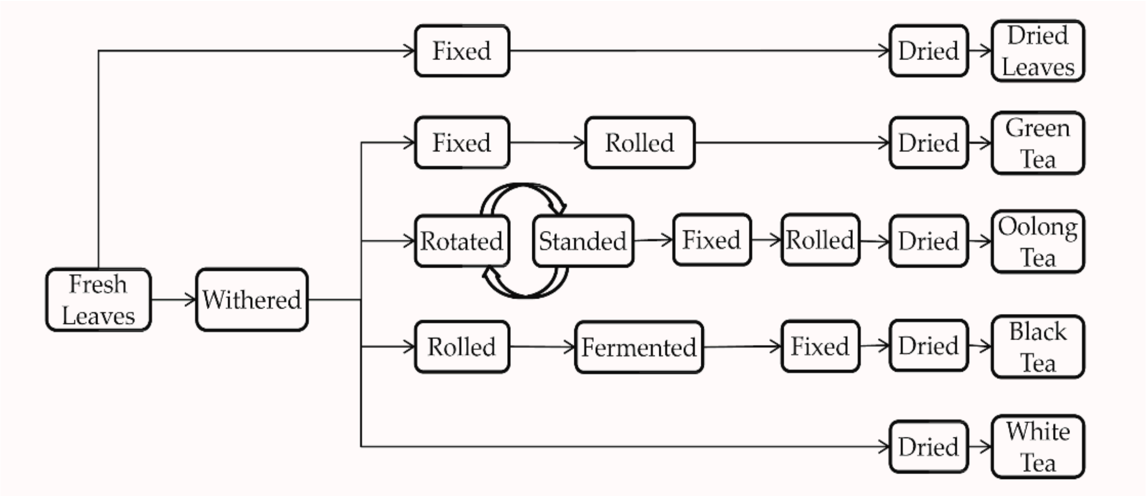

Figure S1. The main manufacturing processes of dried leaves, green tea, oolong tea, black tea and white tea.

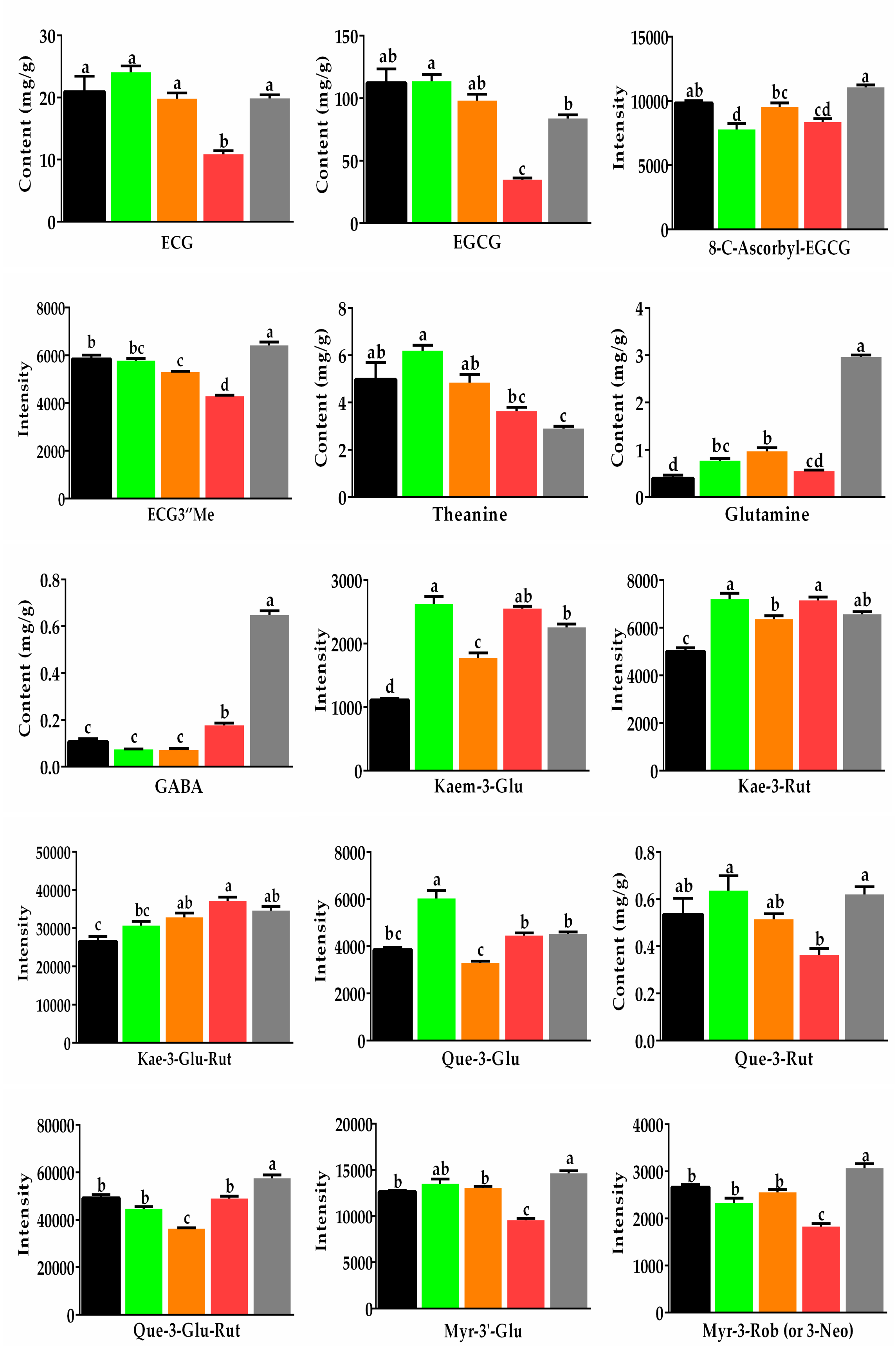
Content (mg/g)
30 20 10 0
a a a b a
ECG
Content (mg/g)
150 100 50 0
ab a ab c b
EGCG
Intensity
15000 10000 5000 0
ab d bc cd a
8-C-Ascorbyl-EGCG
Intensity
8000 6000 4000 2000 0
b bc c d a
ECG3″Me
Content (mg/g)
8 6 4 2 0
ab a ab bc c
Theanine
Content (mg/g)
4 3 2 1 0
d bc b cd a
Glutamine
Content (mg/g)
0.8 0.6 0.4 0.2 0.0
c c c b a
GABA
Intensity
3000 2000 1000 0
d a c ab b
Kaem-3-Glu
Intensity
8000 6000 4000 2000 0
c a b a ab
Kae-3-Rut
Intensity
50000 40000 30000 20000 10000 0
c bc ab a ab
Kae-3-Glu-Rut
Intensity
8000 6000 4000 2000 0
bc a c b b
Que-3-Glu
Content (mg/g)
0.8 0.6 0.4 0.2 0.0
ab a ab b a
Que-3-Rut
Intensity
80000 60000 40000 20000 0
b b c b a
Que-3-Glu-Rut
Intensity
20000 15000 10000 5000 0
b ab b c a
Myr-3'-Glu
Intensity
4000 3000 2000 1000 0
b b b c a
Myr-3-Rob (or 3-Neo)

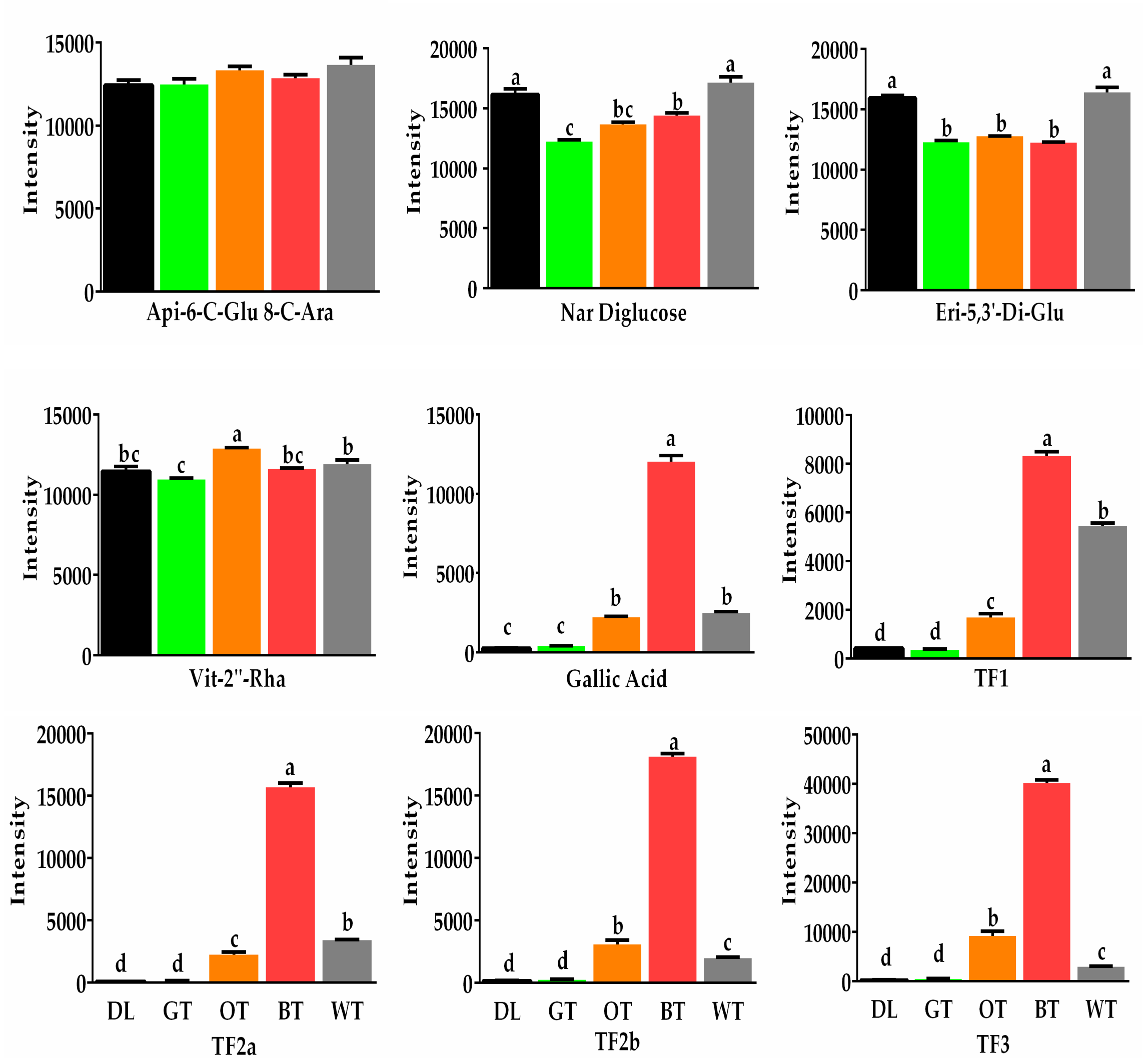

Figure S2. Abundance of precedent or potential neuroprotective compounds among different tea types. Lower case letters indicate significant difference ($p < 0.05$) amongst different samples. Results were expressed as mean ± standard deviation ($n = 3$).
